# An Experimental Workflow for Studying Barrier Integrity, Permeability, and Tight Junction Composition and Localization in a Single Endothelial Cell Monolayer: Proof of Concept

**DOI:** 10.3390/ijms22158178

**Published:** 2021-07-30

**Authors:** Maria Bartosova, David Ridinger, Iva Marinovic, Jana Heigwer, Conghui Zhang, Eszter Levai, Jens H. Westhoff, Franz Schaefer, Stefan Terjung, Georg Hildenbrand, Damir Krunic, Felix Bestvater, Michael Hausmann, Claus Peter Schmitt, Sotirios G. Zarogiannis

**Affiliations:** 1 Center for Pediatric and Adolescent Medicine, University Hospital Heidelberg, 69120 Heidelberg, Germany; Maria.Bartosova@med.uni-heidelberg.de (M.B.); Iva.Marinovic@med.uni-heidelberg.de (I.M.); Jana.Heigwer@med.uni-heidelberg.de (J.H.); Conghui.Zhang@med.uni-heidelberg.de (C.Z.); Eszter.Levai@med.uni-heidelberg.de (E.L.); Jens.Westhoff@med.uni-heidelberg.de (J.H.W.); Franz.Schaefer@med.uni-heidelberg.de (F.S.); ClausPeter.Schmitt@med.uni-heidelberg.de (C.P.S.); 2 Kirchhoff Institute for Physics, Heidelberg University, 69120 Heidelberg, Germany; d.ridinger@mailbox.org (D.R.); hilden@kip.uni-heidelberg.de (G.H.); hausmann@kip.uni-heidelberg.de (M.H.); 3 1st Department of Pediatrics, Semmelweis University, 1083 Budapest, Hungary; 4 ELKH-SE Pediatrics and Nephrology Research Group, 1083 Budapest, Hungary; 5 European Molecular Biology Laboratory, Advanced Light Microscopy Facility, 69117 Heidelberg, Germany; terjung@embl.de; 6 German Cancer Research Center, 69120 Heidelberg, Germany; d.krunic@dkfz.de (D.K.); f.bestvater@dkfz.de (F.B.); 7 Department of Physiology, Faculty of Medicine, School of Health Sciences, University of Thessaly, BIOPOLIS, 41500 Larissa, Greece

**Keywords:** Alanyl-Glutamine, automated immunofluorescence imaging, cell monolayer, claudins, endothelial cells, paracellular permeability, single molecule localization microscopy, tight junctions, Transwell, Zonula Occludens-1

## Abstract

Endothelial and epithelial barrier function is crucial for the maintenance of physiological processes. The barrier paracellular permeability depends on the composition and spatial distribution of the cell-to-cell tight junctions (TJ). Here, we provide an experimental workflow that yields several layers of physiological data in the setting of a single endothelial cell monolayer. Human umbilical vein endothelial cells were grown on Transwell filters. Transendothelial electrical resistance (TER) and 10 kDa FITC dextran flux were measured using Alanyl-Glutamine (AlaGln) as a paracellular barrier modulator. Single monolayers were immunolabelled for Zonula Occludens-1 (ZO-1) and Claudin-5 (CLDN5) and used for automated immunofluorescence imaging. Finally, the same monolayers were used for single molecule localization microscopy (SMLM) of ZO-1 and CLDN5 at the nanoscale for spatial clustering analysis. The TER increased and the paracellular dextran flux decreased after the application of AlaGln and these functional changes of the monolayer were mediated by an increase in the ZO-1 and CLDN5 abundance in the cell–cell interface. At the nanoscale level, the functional and protein abundance data were accompanied by non-random increased clustering of CLDN5. Our experimental workflow provides multiple data from a single monolayer and has wide applicability in the setting of paracellular studies in endothelia and epithelia.

## 1. Introduction

The study of epithelial and endothelial barrier permeability is critical for understanding basic physiological functions that are seminal for sustaining physiological cell and organ function. Processes like the reabsorption and secretion of water, ions, and nutrients in the nephron and gastrointestinal tract, as well as the filtration of plasma in the areas of capillary vascular beds, rely on the proper function of the associated epithelial and endothelial barriers [1]. The permeability characteristics of such barriers are defined by the transcellular and paracellular route of water, small molecules, and ion transport [2]. 

Transcellular permeability necessitates the function of the basolateral Na^+^-K^+^-ATPase that sustains low intracellular Na^+^ concentrations (around 10 mM) and thus maintains an electrochemical gradient between the extracellular and the intracellular compartments allowing for the function of apically located ion channels, cotransporters, and antiporters that mediate ion, water, and small molecule absorption and secretion [3]. This process also requires the polarization of the epithelial and endothelial monolayers that is ensured by the function of tight junctions (TJ) [4]. Through their “fence” function, TJ ensure that no lateral diffusion of transmembrane proteins, e.g., ion channels, transporters, or pumps, can occur from the apical to the basolateral side of the cell membrane (and vice versa), thus maintaining an asymmetry of distribution of such molecules and along with the function of Na^+^-K^+^-ATPase, a potential difference between the two sides of the monolayer [5]. This implies that the paracellular molecular machinery is essential for proper epithelial and endothelial barrier function. 

TJs are the molecular counterparts of the paracellular barrier formation and consist of claudins (a protein family with 27 members), junctional adhesion molecules (JAMs), and accessory proteins that are located in the intracellular compartment of TJ and mediate the connection of TJ with the actin cytoskeleton. Zonula occludens proteins (ZO1-3) are the most important ones [6]. Depending on the composition of the TJ in terms of Claudins and JAMs, a barrier can be variably permeable to water, ions and small molecules [7]. The paracellular route can be divided in two pathways, namely the pore pathway that modulates the passage of ions and water in a charge and size selective manner and the leak pathway that modulates the passage of ions and small molecules in charge and size non-selective manner [8]. At the ultrastructural level, the TJ comprise a highly ordered, dynamic in nature, belt-like meshwork of anastomosing strands that encircle cells at the proximal apical membrane side [6]. Changes in the TJ composition (i.e., stoichiometry of claudin types in the complex) are also accompanied by re-organization at the ultrastructural level [9,10,11,12]. Pro-inflammatory cytokines and reactive oxygen species are modulators of the TJ [13,14,15,16,17]. 

The current methodological approach to study the paracellular permeability in vitro involves the development of cell monolayers on Transwell porous filters by monitoring the electrical resistance until a plateau is reached (i.e., monolayer is confluent). Subsequently, chemical stimuli are applied and then the resistance changes are measured as a function of time. Increase in the resistance implies that the monolayer becomes tighter, and a less ionic current can pass paracellularly [18,19]. With the same setup, a fluorescently labelled dextran can be added on the apical compartment and its leakage into the basolateral compartment can be measured at given time points, in order to assess changes in the small molecule paracellular permeability [20]. Different sizes of dextrans (4 kDa to 2000 kDa) can provide evidence regarding the increase or decrease in the leakage of small molecules and macromolecules. Although this approach provides important information, it does not provide any evidence regarding the paracellular pathway changes on the molecular level. This is achieved when, at the end of an experiment as described above, ordinary or confocal microscopy immunofluorescence is performed on the filter probing with antibodies against specific claudin or ZO proteins [21,22]. Moreover, there is still no information provided regarding the ultrastructural organization of TJ. In most cases, these data have been produced when the monolayers are subjected to transmission electron microscopy (TEM) or freeze fracture electron microscopy (FFEM) in order to visualize the ultrastructural morphological changes in the interface of adjacent cells with high resolution, but in this case an evaluation of specific TJ proteins is not possible as a totally different sample preparation procedure is required [23,24]. Recent advances in single molecule localization microscopy (SMLM) have provided opportunities for a more in-depth assessment of TJ changes with high spatial resolution as recently illustrated in studies involving ZO-1 in human endothelial cells [25]. This approach allows for the use of monolayers grown in filters after being subjected to TER, dextran leakage measurements, and immunostaining for specific TJ molecules [25,26]. Combining these methods in one experimental setting should circumvent inter-experimental variations and allow for combined analyses of specific TJ expression and localization, functional studies, and single molecule cluster analyses in a matched manner.

In this paper we provide an experimental workflow that combines functional studies (TER and dextran permeability measurements) with immunofluorescence and with SMLM probing for ZO-1 and CLDN5 in the same human endothelial cell monolayer. To provide proof of the concept, we treated the endothelial cells with Alanyl-Glutamine (AlaGln), a dipeptide that tightens the paracellular barrier [25]. 

## 2. Results

### 2.1. Transendothelial Resistance and Paracellular Dextran Permeability Assay

**Figure 1:**
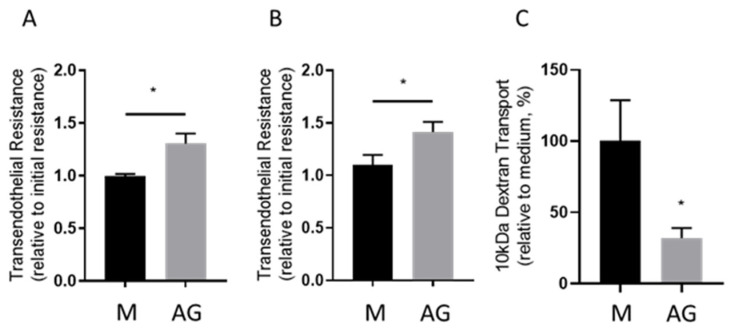
Functional endothelial cell monolayer characterization under the influence of 24 mM AlaGln (AG). The transendothelial resistance (TER) was measured at one (**A**) and five (**B**) hours of AlaGln incubation. FITC-dextran (10 kDa) was introduced apically at time 1 h post AG incubation and its transport to the basolateral compartment was measured after 5 h (**C**). Incubation with 24 mM AG resulted in increase of TER after 1 and 5 h, while the 10 kDa FITC dextran transport was reduced. M = control medium. Data are presented as mean ± SD, *n* ≥ 6, * *p* < 0.05.

Primary human umbilical vein endothelial cells (HUVEC) were seeded on polyester (PE) or polycarbonate (PC) Transwell filters. In parallel, HUVEC in the same plating density were seeded on coverglasses placed in a 12-well cell culture plate in order to be used in the subsequent immunocytofluorescence steps along with the filters. While values of the blank Transwell filters (filters without cells being bathed by cell culture medium only) differed between PE (121 ± 4 Ω) vs. PC (88 ± 5 Ω, *p* < 0.001) filters, the resulting baseline resistance reached by the HUVECs after correction for the blank value was similar (12.9 ± 3.5 vs. 13.2 ± 1.6 Ω cm^2^; *p* = 0.85 in PE vs. PC filters). After cells have formed a confluent monolayer, baseline TER was measured and medium was exchanged for treatment solution. We incubated HUVECs with 24 mM AlaGln, known to induce the tightening of endothelial barriers [25]. After one hour, TER was measured again and the treatment solutions were exchanged to contain FITC labeled dextran (10 kDa) and incubated for four hours. HUVECs grown on coverglasses were also treated with AlaGln for five hours. After one hour of incubation with medium supplemented with 24 mM AlaGln, transendothelial resistance of HUVEC monolayers increased by a factor of 1.30 ± 0.22 (i.e., TER_t_/TER_INITIAL_) relative cells incubated with medium only (*p* = 0.013; Figure 1A). After five hours, the increase was similar (1.41 ± 0.21 compared to control (medium incubated cells, *p* = 0.049; Figure 1B). After the one-hour time point, 10 kDa FITC dextran was added to the apical medium of the Transwell and the transport to the basolateral compartment was measured four hours later. In AlaGln exposed cell monolayers, 10 kDa FITC dextran concentration in the basolateral compartment was reduced to 31.7 ± 0.07% in comparison to control cells (*p* = 0.016; Figure 1C). These results demonstrate that AlaGln tightens the paracellular barrier. 

### 2.2. Immunofluorescence Probing for ZO-1 and CLDN5

Co-staining of ZO-1 and CLDN5 was performed on the same Transwell filters used for the functional analysis. Monoclonal antibodies conjugated to secondary antibody (Alexa 647, 555 or 488) or polyclonal antibodies stained with secondary Alexa-antibody against the host species of the primary antibody were used. Alexa 488 conjugated antibodies were excluded, because of the highest autofluorescence background signal of the filter at 488 nm excitation. Filters were cut out from the Transwell plastic and mounted a glass slide. Controls and treatment conditions were mounted on the same glass slide to ensure equal microscopy settings. Coverglasses were mounted in the glass slides likewise. Automated, z-stacks based imaging of filters/coverglasses was performed on the ACQUIFER Imaging Machine. Images were acquired for all channels and were processed afterwards with specialized software (see Methods).

**Figure 2:**
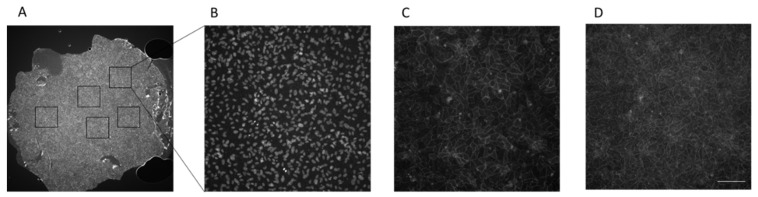
Automated imaging of the filter. Endothelial cells either grown on polycarbonate (PC) Transwell filter, polyester (PE) Transwell filter or on glass slide were stained and analyzed by automated imaging. First, the whole filter/slide area was imaged in 2× magnification. 4′,6-Diamidin-2-phenylindol (DAPI) staining was used to find focal plane (**A**). 5 randomly selected areas (squares) were further imaged with a 20× objective with excitation wavelengths of 385 nm (**B**, **DAPI**), 555 nm (**C**, **Alexa 555**) and 647 nm (**D**, **Alexa 647**). Grey scale pictures from PC filter are shown for all channels. Scale bar = 100 µm.

As shown in Figure 2, after imaging of the whole area at 2× magnification, five randomly chosen regions (representing 15% of the whole filter area) were automatically imaged with 20× objective using the same specifications (illumination power and exposure time). 

Ten z-stacks were acquired (image distance 3 µm), and maximum intensity projection was carried out prior to analysis. Fluorescence intensity was quantified on grey scale images with Fiji, and at least 10 random junction areas per image were analyzed. The analysis was repeated with the complete filter area. For this purpose, 100 images with 10% overlap were obtained and stitched together with an in-house stitching script [27]. 

**Figure 3:**
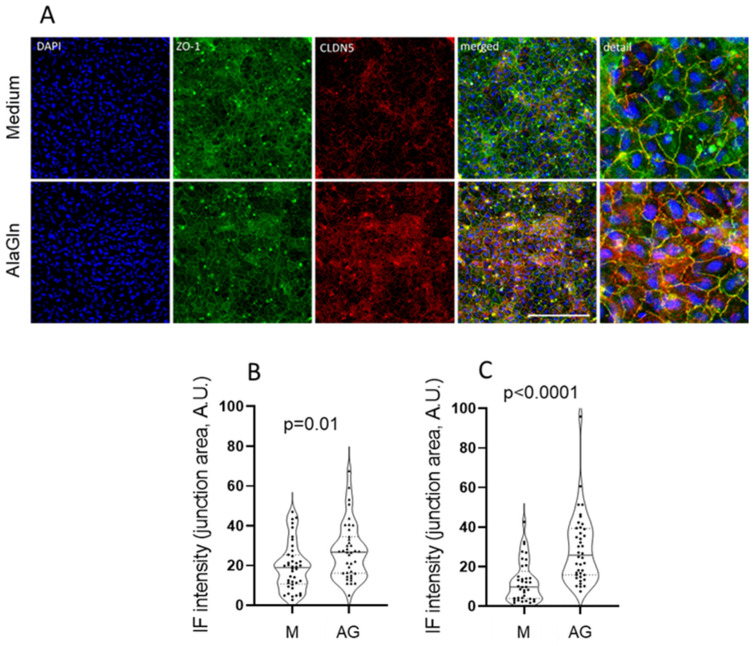
Visualization and quantification of tight junction protein. Immunocytochemical staining of Zonula Occludens-1 (ZO-1) and claudin-5 (CLDN5) in HUVEC monolayers on the same PC filters that were used for the TER and paracellular dextran permeability functional studies. In the upper row medium incubated HUVECs are shown and in the lower row HUVECs incubated with 24 mM AlaGln (AG) are shown (**A**). Immunofluorescence of ZO-1 and CLDN5 in the junction areas was quantified (z-stack spacing 3 µm). Scale bar = 100 µm. The quantification of AG treatment vs. medium control for ZO-1 is shown in (**B**) and for CDLN5 in (**C**).

Treatment with AlaGln increased ZO-1 (27.3 ± 14 vs. 19.5 ± 12 A.U. with medium control; *p* = 0.01) and CLDN5 intensities (29 ± 17 vs. 12.4 ± 10 A.U.; *p* < 0.0001) at the junction areas (Figure 3). Comparable differences between AlaGln and medium treated monolayers were obtained when the whole filter area was analyzed (ZO-1 intensity 0.63 ± 0.29 A.U. after 24 mM AlaGln exposure vs. 0.51 ± 0.23 A.U. in medium; *p* = 0.04 and CLDN5 0.68 ± 0.25 A.U. vs. 0.44 ± 0.33 A.U. in medium only; *p* = 0.007). The results did not differ between PE and PC filters. 

HUVEC grown on coverglasses showed a similar tendency towards increase in junctional ZO-1 with AlaGln (26.3 ± 14 vs. 19.7 ± 11 A.U. with medium, *p* = 0.07). However, CLDN5 was not different (20.3 ± 11 vs. 18.0 ± 10 A.U., *p* = 0.48). Thus, in PC filters, a better discriminatory capacity was probably due to better polarization of the monolayer was achieved. 

Next, we analyzed in how far staining results were influenced by characteristics of the antibodies. While ZO-1 was available as monoclonal Alexa conjugated antibody detectable in several excitation wavelengths (488, 555, and 647 nm), CLDN5 antibody was only available conjugated to Alexa 488. While ZO-1 abundance changes after the incubation with 24 mM AlaGln were comparable, independent of the antibody and conjugate used, the corresponding CLDN5 changes were only detected with the monoclonal antibody at 555 nm, but not the polyclonal antibody at 647 nm (Table 1). Antibodies labelled with Alexa 488 did not prove suitable for localization microscopy in preliminary experiment and were therefore not used further.

### 2.3. SMLM for Localization of ZO-1 and CLDN5 Clustering

TJ function depends on the spatial organization of the belt-like structures, thus abundance measurements with immunofluorescence are only partially informative [7]. Therefore, following the automated imaging described above, the same filters (cell monolayers) were further analyzed by SMLM [25,28]. Due to the intrinsic property of Transwell filters to exhibit higher background autofluorescence, we repeated the analysis with HUVECs grown on coverglasses in parallel. The imaging results were comparable with those for IF and SMLM imaging. Coverglasses had the least background, followed by polycarbonate (PC) and polyester (PE) filters. 

Single molecule quantification and cluster analysis was performed separately for each channel. Two critical observations for the optimal outcome of the experiments were that (a) the combination of ZO-1 555 and CLDN5 647 antibodies did not produce a sufficient number of blinking events, therefore monoclonal antibodies against ZO-1 647 and CLDN5 555 were used for the SMLM and (b) double channel imaging was not possible in case of PE filters, because of the high autofluorescence background. In AlaGln treated monolayers (1.30 × 10^−4^ ± 0.11 × 10^−4^) ZO-1 molecules/nm^2^ were counted compared to (1.20 × 10^−4^ ± 0.09 × 10^−4^) in monolayers incubated with medium only (*p* = 0.242). CLDN5 counts were (2.23 × 10^−5^ ± 0.33 × 10^−5^) molecules/nm^2^ in AlaGln treated vs. (8.56 × 10^−5^ ± 0.62 × 10^−5^) molecules/nm^2^ in medium treated cells (*p* < 0.0001). Depending on the size and structure of the individual TJ, ROIs of different sizes were selected to match the TJ form as closely as possible. Therefore, comparing absolute fluorophore count values is not valid in this case and count values were normalized for area for comparisons.

**Figure 4:**
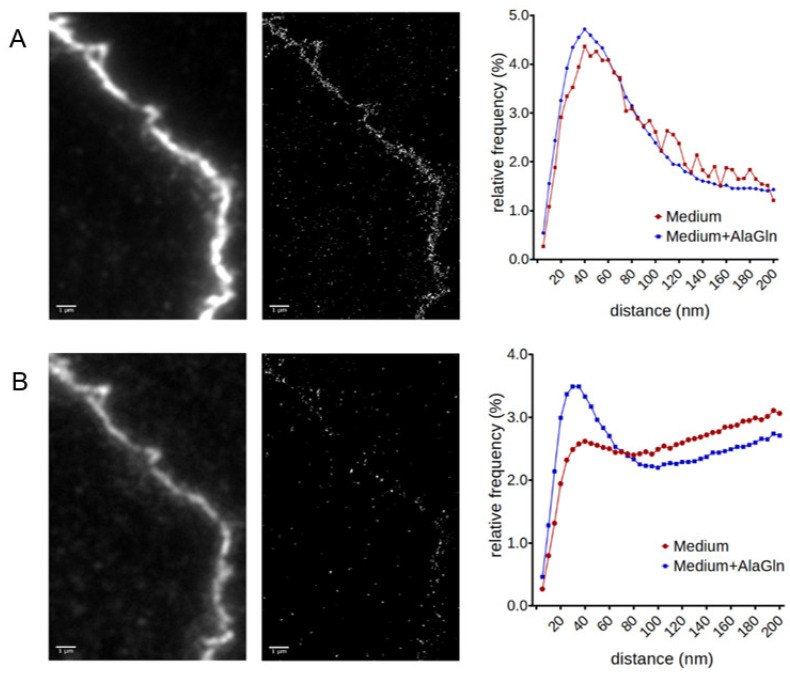
Cluster analysis following single molecule localization microscopy. ZO-1 (**A**) shows comparable frequency of clusters at 40 nm after AlaGln treatment, while clustering it was increased for CLDN5 (**B**) compared to medium treated HUVEC. Left: Widefield images of contact zones. Scale bar = 1µm. Middle: Pointillist images of contact zones created by SMLM Right: Ripley’s pairwise distance frequency histograms indicating cluster formation (peak at smaller distances) in a dispersed environment.

SMLM of HUVEC monolayers on the PC filters revealed increased clustering of CLDN5 molecules after five-hour treatment with 24 mM AlaGln, while no significant change was observed for ZO-1 (Figure 4). In the presence of AlaGln, the relative frequency of CLDN5 molecule clusters was significantly higher in the range of 40 nm, which suggests a more organized and less permeable TJ belt, as CLDN5 is a known endothelial barrier protein. This corroborates the findings of the functional analysis in the Transwell monolayer (TER and dextran flux) as well as the immunocytofluorescence findings of more CLDN5 being present at the junctional interface of the cell–cell contacts. The AlaGln induced increased clustering, which was similar when the analysis performed was limited to the cell junction area only and when glass slides were analyzed. When PE filters were used, no differences in the clustering of ZO-1 and CLDN5 were detected, most likely due to the high background autofluorescence of the PE filter reducing the sensitivity of the method.

**Figure 5:**
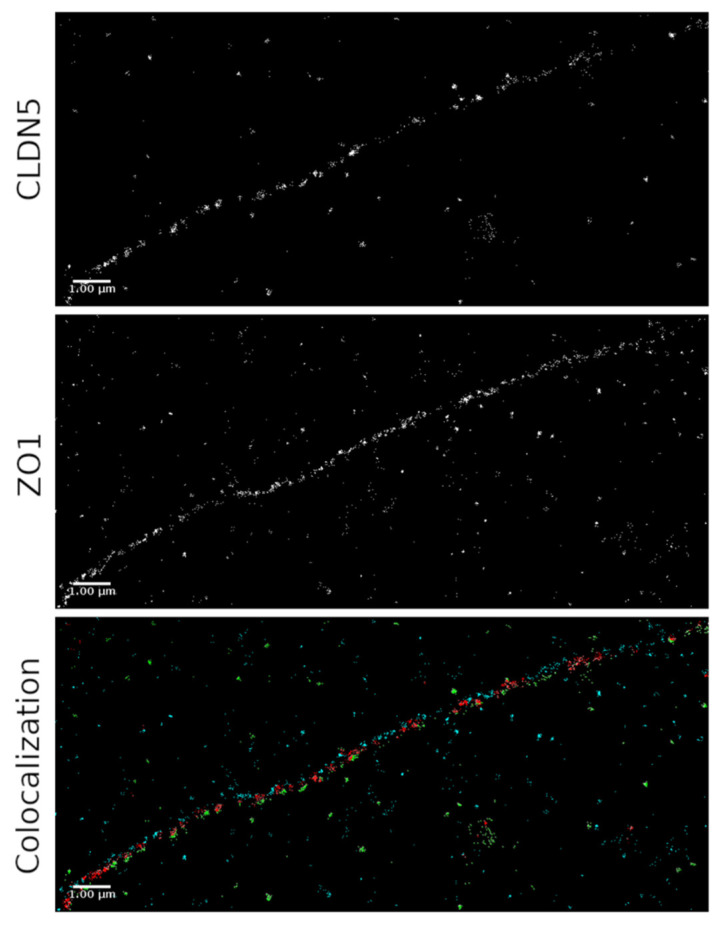
Colocalization analysis of ZO-1 and CLDN5 performed by SMLM. ZO-1 molecules are shown in blue, CLDN5 molecules in green, and co-localized molecules within 90 nm of each other are shown in red.

Immunofluorescence images are randomly selected and cover a large area of the filter, but provide information on the overlapping of ZO-1 and CLDN5 with limited localization precision and resolution only. SMLM colocalization studies demonstrated the single, distinct ZO-1 and CLDN5 molecules, and their precise spatial distribution and colocalization (Figure 5). Colocalization was defined as the distance of fluorophores of different wavelengths within 90 nm. This range considers the size of two antibodies with fluorophores and their possible minimal distance. To determine the spatial organization of ZO-1 (shown in blue) and CLDN5 (shown in green), the distances between individual ZO-1 molecules and individual CLDN5 molecules were quantified up to a distance of 400 nm. Ripley’s pairwise distance frequency histograms indicated an organized clustering of the colocalized ZO-1 and CLDN5 molecules. Their distribution however, was not affected by AlaGln supplementation (Supplementary Figure S1). The amount of colocalized molecules was quantified in both ZO-1 and CLDN5 channels. The percentage of CLDN5 molecules co-localized to ZO-1 was (42.4 ± 4.7)% in AlaGln treated cell compared to (46.1 ± 6.5)% in medium treated monolayers (*p* < 0.001). The percentage of ZO-1 molecules co-localized to CLDN5 was (11.4 ± 1.5)% in AlaGln treated cells vs. (25.4 ± 3.8)% in medium treated cells (*p* = 0.160), reflecting the much higher abundance in CLDN5 than ZO-1 in response to AlaGln (Figure 5). 

### 2.4. Integration of the Multiple Stepwise Tight Junction Analyses of a Single Experiment

**Figure 6:**
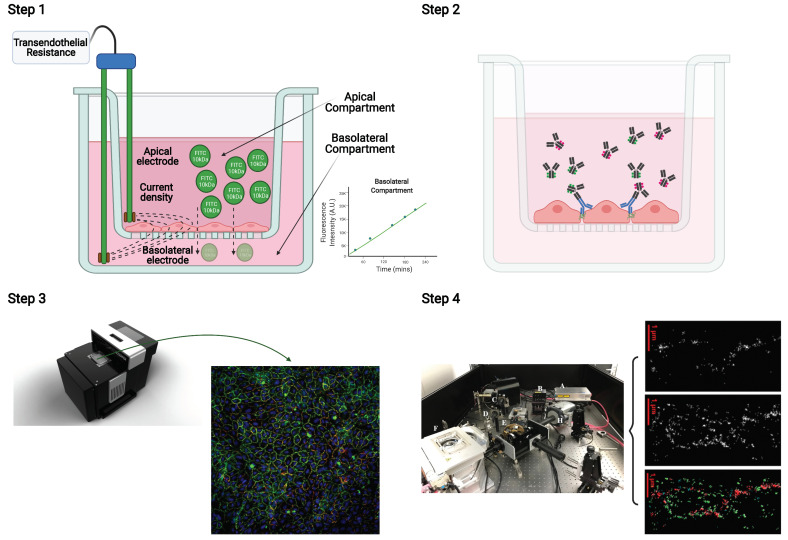
Integration of the stepwise experimental workflow for in-depth TJ study in endothelial cells. HUVECs were grown on PC Transwell filters, and the transendothelial resistance (TER) of the monolayer was measured with a volt/ohm meter until confluence. (**Step 1**): After confluent monolayer formation, experimental solutions were added and TER changes along with 10kDa FITC dextran paracellular transport were monitored for a 4-hour period. (**Step 2**): Subsequent immunofluorescent staining was performed on the monolayer in the PC Transwell filter with different fluorescently labeled antibodies. (**Step 3**): Automated immunofluorescence intensity analysis was performed from a z-stack by the ACQUIFER Imaging Machine comprising 10% of the entire insert. (**Step 4**): The same filter was subjected to single molecule localization microscopy (SMLM) followed by image processing of the junction area. Image processing algorithms were employed in order to assess the clustering of the TJ proteins of interest. The Figure was constructed with BioRender.com (accessed on 17 March 2021).

The four-step experimental workflow performed on the same Transwell filter is summarized in Figure 6. Primary HUVECs were seeded on PC Transwell filters and monitored until a confluent monolayer with stable TER was achieved. Then, the specific intervention was introduced (addition of 24 mM AlaGln containing medium) and the changes in TER were monitored along with the paracellular flux of a fluorescent dextran as a function of time. Thereafter, the same monolayers were fixed and probed with appropriate antibodies for immunocytofluorescence against the TJ components ZO-1 and CLDN5 and changes in the according fluorescence intensity were recorded by automated z-stack fluorescence analysis of the 10% of the monolayer. Spatial clustering and colocalization of the components of the TJ under study were then analyzed by SMLM in the same filter. 

## 3. Discussion

Endothelial cells form the inner monolayer vesting the vessels throughout the cardiovascular system and provide a well-regulated barrier, important for the provision of the underlying tissues with oxygen, nutrients, and for the removal of cell metabolites and toxins [29]. The polarization of endothelial cell monolayers is critical for their absorptive and/or secretory functions as well as the prevention of pathogens entering the systemic circulation [30,31]. The tight junctions are a dynamic structure that can be reorganized depending on the external stimuli in a way that the paracellular permeability can adapt to the required characteristics of diffusion restriction [32]. The way claudins interact with other TJ machinery components such as the zonula occludens (ZO) adaptor proteins is an area of great scientific interest and requires methodologies providing functional analyses together with high spatial and temporal resolution [6]. In several pathological conditions, like anaphylaxis or sepsis, the disruption of the endothelial TJ is a hallmark of disease severity and progression since the leakage of proteins to third spaces will follow and induce interstitial and organ oedema that can be fatal for the patient [33,34,35]. Therefore, a thorough understanding of the mechanisms underlying this disruption and the investigation of potentially therapeutic compounds is of great interest. 

In the current study, we have presented an experimental workflow that allows for the in-depth and comprehensive study of the paracellular pathway properties from a single endothelial monolayer. We have combined TER, paracellular dextran flux, immunofluorescence for TJ abundance estimation, and SMLM for the assessment of TJ spatial organization profile in endothelial cells. In this context, we chose to investigate the important endothelial TJ components CLDN5 and ZO-1. In order to demonstrate the validity of our findings, we have used AlaGln, a dipeptide that has been reported to seal the TJ in endothelial cells [25]. Indeed, under the effect of AlaGln, it was shown that the TER increased along with a concomitant decrease in the paracellular flux of 10 kDa FITC dextran at 4 h post incubation. Another layer of information was provided by means of automated immunocytofluorescence analysis, showing that this effect was mediated by the increase in the fluorescent intensity of both ZO-1 and CLDN5 in the junctional areas. Altogether, these data indicated that AlaGln tightened the endothelial barrier. The subsequent SMLM analysis demonstrated that this phenotype was not only a result of higher ZO-1 and CLDN5 abundance, but a key for functional properties, also a result of the enhanced clustering of CLDN5 in the cell–cell area. Thus, our approach can provide a functional, temporal, and spatial description of the paracellular permeability changes that occur in the TJ under the influence of a certain stimulus in the context of one single monolayer. 

Currently, the methodological approaches for assessing the paracellular permeability of an epithelial or endothelial monolayer mainly involve the measurement of TER and paracellular fluorescently labelled dextran fluxes [18,19,20]. These studies provide information concerning the ionic current passing through the monolayer and its leakage to macromolecules. However, no information on the underlying molecular mechanisms of these changes can be provided. For such change, Western blot or immunofluorescence studies can be performed [21,22]. The first approach provides information on the global abundance of a TJ protein in the cell monolayer, which can be narrowed by blotting the cell membrane fraction of the monolayer only. This provides a quantitative measure of the specific cell-to-cell changes of the monolayer, but it is a laborious procedure that requires a large number of cells [36]. Immunofluorescence provides similar results with the same cell membrane fraction as Western blotting, as well as a better visual understanding of the associated changes. A better description of the localization and associated changes of the TJ proteins could be acquired by confocal microscopy performed on the monolayer in the Transwell filter [22]. Automated imaging provides the possibility to analyse large amounts of samples simultaneously and is time-efficient compared to other imaging techniques. Moreover, confocal microscopy, which is a standard imaging method in TJ analysis, providing Z stack-based analysis covering the whole range from basolateral to apical sides of the cell, may result in fluorophore bleaching. Thus, imaged monolayers cannot be used for further analysis like SMLM due to the higher laser power needed for visualization [37]. For the generation of data at the ultrastructural level of TJ, either TEM or FFEM is employed, but the evaluation of specific TJ molecules is not possible as a totally different sample preparation procedure is required [23,24]. Recent advances in SMLM have provided opportunities for a more detailed assessment of the TJ changes with high spatial resolution, as we have recently demonstrated in studies involving ZO-1 in human endothelial cells [25]. In such a case, the standard preparation for immunofluorescence on the monolayer is required allowing for the serial combination of different techniques prior to the SMLM experiment [26]. This approach, therefore, allows for the use of a single monolayer grown on Transwell filters after being subjected to TER, dextran leakage measurements, and immunocytofluorescence staining for specific TJ molecules, to be used for the evaluation of the TJ changes on the nanoscale. 

Our experimental workflow provides several advantages. Fewer experiments are needed to collect all the relevant results, and this way the variability among experiments using two or three different monolayers is eliminated, and powerful paired statistical analysis can be performed [38,39]. The issue of reproducibility in life sciences has long been debated and adopting approaches that yield several experimental results from a single experimental setup leads to more robust results and a reduction in the associated costs. Primary cells are usually preferred, but this imposes a pressing need for performing experiments within the first few passages to prevent bias by cell dedifferentiation [40,41]. The application of our experimental workflow that increases the output of results is of considerable advantage. On the same note, as the 3R (replacement, reduction and refinement) principle is further adopted in research laboratories, our experimental workflow can be an asset in studies where primary cell cultures are derived from animal models of disease [42]. Lastly, our experimental workflow is widely applicable in practically all cell types that form monolayers, and paracellular permeability and TJ study is of value, e.g., in epithelial, mesothelial, and endothelial cells [43]. The fact that PC filters were shown to be suitable for this workflow suggests that, with small modification, this workflow could be used in Ussing chamber experiments where Snapwell PC filters are used. 

The limitations of the proposed experimental workflow include the fact that it is currently challenging to obtain access to automated immunofluorescent imaging systems like ACQUIFER and to SMLM facilities. However, these technologies will be progressively more accessible. A future challenge is to broaden the possibilities of the automated immunofluorescence and SMLM analyses, e.g., to three TJ components studied in terms of colocalization. 

## 4. Materials and Methods

### 4.1. Cell Culture

Human umbilical vein endothelial cells (HUVEC) were commercially purchased (PromoCell, Heidelberg, Germany) and kept in endothelial cell growth medium with supplement and antibiotics (PromoCell, Heidelberg, Germany) in an incubator at 37 °C and 5% CO_2_. All experiments were performed on cells within the first 5 passages. 

### 4.2. Trans-Endothelial Resistance

To establish a model of HUVEC monolayers in vitro, a cell suspension (5 × 10^4^ cells/cm^2^) was seeded and cultured on a polyester/polycarbonate mesh (Transwell, 0.4 µm pore size, 12-well type; Costar, MA, USA) using standard culture conditions. The apical and basolateral chambers of the Transwell were filled with 0.2 mL and 1 mL culture medium, respectively. Transendothelial electrical resistance (TER) was measured daily using an EVOM volt ohm meter equipped with STX-2 electrodes (World Precision Instruments, Sarasota, FL, USA). The electrodes were inserted into both ends of the mesh. An alternating current of less than ±20 µA was applied between the electrodes at a frequency of 12.5 Hz. To calculate the normalized TER of each monolayer, the background TER of a blank filter was subtracted from the TER of the respective cell monolayer. The resistance of each monolayer was multiplied by the effective surface area (0.33 cm^2^) corresponding to the filter size in order to obtain the electrical resistance of that monolayer (in Ω·cm^2^). The treatment was initiated when each monolayer was fully formed as demonstrated by a plateau in the TER (4–6 days post-seeding), and the baseline TER was >10 Ω·cm^2^. The data are presented as % fold change of the cells cultured in standard culture conditions (control).

### 4.3. Paracellular Endothelial Barrier Dextran Transport Assessment

The paracellular permeability of the HUVEC monolayers was determined by measuring the flux of 10 kDa fluorescein isothiocyanate (FITC) labelled dextran (obtained from Sigma Aldrich, Taufkirchen, Germany) from the apical to the basolateral compartment of a Transwell chamber as a function of time. More specifically, 1 mg/mL was added in the apical compartment of a Transwell chamber and the increase of the fluorescence intensity in the basolateral Transwell compartment after 4 h. An equimolar amount of unlabeled dextran was added to the basolateral compartment of the transwell system to maintain an isotonic condition. At 4 h after the addition of the 10 kDa FITC-dextran, a 10 µL volume of each sample was collected from both sides of the chamber. Each sample was assessed using a fluorescence spectrophotometer (F-2000; Hitachi, Tokyo, Japan) at an excitation wavelength of 490 nm and an emission wavelength of 520 nm. A calibration curve was established and used for the calculation of the amount of FITC dextran, which was transported to the lower compartment. Results are presented as % fold change of the cells cultured in standard culture conditions (control).

### 4.4. Immunostaining

Coverglasses/filters were fixed in absolute ethanol at −20 °C for 5 min, washed, permeabilized (0.5% Triton X in PBS for 10 min), washed again and blocked (5% bovine serum albumin in PBS) for 1 h at room temperature (RT). Incubation with the primary antibody was performed overnight at 4 °C. The appropriate secondary fluorescent antibody was added on the next day for overnight at 4 °C. For double-staining, coverglasses/filters were fixed again with 4% PFA for 20 min at RT, washed and incubated again with the primary antibody. Polyclonal antibodies were incubated first. Nuclei were stained with DAPI (1:1000). After washing, the filters were cut out from the plastic by a needle tip, put on glass slide, covered with Prolong Gold (Thermo Fischer Scientific, Dreieich, Germany) and let harden at least for 24 h at RT in the dark and kept at 4 °C until analysis. PBS (1.25 mM Ca^++^and 1.75 mM Mg^++^ was used to stabilize the cell membranes throughout the staining procedure.

### 4.5. Automated Imaging and Image Analysis

Fully developed HUVEC monolayers were used for automated immunofluorescence imaging of ZO-1 and CLDN5 on an ACQUIFER Imaging Machine, a widefield high-content screening microscope (ACQUIFER Imaging GmbH, Heidelberg, Germany). The Imaging Machine is equipped with NIKON objectives, a white light emitting diode (LED) array for brightfield imaging combined with a LED fluorescence excitation source, a HAMAMATSU sCMOS camera (resolution 2048 × 2028 pixels), and an immobile microplate slot with a temperature controller. A key feature is the moving optics that are software controlled and allow for precision imaging with no platform drift. The focal plane was detected in the 4′,6-Diamidin-2-phenylindol (DAPI) channel (385 nm) using a built-in software autofocus algorithm. Filters and coverglasses were embedded in Prolong Gold and fixed on glass slide. Medium controls and AlaGln treated cells were put on one glass slide for the imaging. 

For each condition z-stack images (10 slices with 3 µm slice distance) using excitation light of 385 nm, 555 nm and 647 nm were acquired using a 20× NA 0.45 objective. The filter emission detection ranges are: 415–480 nm, 580–640 nm, and 660–710 nm. Integration times were fixed at 100% relative LED intensity and 200 ms exposure time for 555 nm and 647 nm channels, and 40% relative LED intensity and 20 ms exposure time for the DAPI channel. 

Post imaging analysis was performed using Fiji software (GPL v2) [44]. Greyscale images were used to create z-stack projections using maximum intensity method to obtain a clear signal from cell membrane areas. Ten stacks were used for every condition. Ten different areas per condition were analyzed, and data are presented as intensity from grey scale image/analyzed area. Secondary analysis was limited to the tight junction areas. For this purpose, cell membranes were annotated manually, while intensity was measured and corrected for analyzed area to obtain a final result. 

### 4.6. Single Molecule Localization Microscopy (SMLM)

For the SMLM experiments, a custom-made apparatus based on an iMic microscope (Till Photonics, FEI) was used [36,45,46]. The SMLM system is equipped with an Acousto-Optical-Tunable-Filter (AOTF), a variable beam expander (Standa Ltd., Vilnius, Lithuania), a Flat-Top-Profile forming optics—PiShaper (AdlOptica GmbH, Berlin, Germany), a 100×/NA 1.46 oil plan apochromatic objective lens (Carl Zeiss Microscopy, Göttingen, Germany) and four lasers: 405, 491, 561, and 642 nm with maximal laser power of 120, 200, 200, and 140 mW, respectively. In our study, 561 nm laser was used at 70% output power (corresponding to 150 mW) and 642 nm laser at 100% output power (140 mW), respectively. The system was maintained free from environmental influences by thermomechanical stabilization (±10^−2^ K), constant monitoring of the temperature in the measurement environment and liquid cooling of the main critical components. The SMLM measurements were initiated after allowing an hour for thermal equilibrium used in order to avoid thermal expansion effects. The fluorescent light was recorded by an iXon Andor Ultra EMCCD camera (Andor Technology, Belfast, Northern Ireland) (80 nm/px, EM-gain set to 100). To allow for comparisons among measurements, the following automatized image acquisition protocol was employed: After a 10 s and 20 s flash at 150 mW and 140 mW, respectively, fluorophores were set into a reversible bleached state. Subsequently, 2000 images (100 ms integration time) were recorded and stored as a 16-bit grey-scale *tiff image stack. In addition to the SMLM data stack, a widefield image of the relevant specimen region was recorded.

### 4.7. SMLM Data Analysis

SMLM data analysis was performed with and in-house developed python-based package and the use of MATLAB software [28,47]. Noise reduction was achieved by the use of a threshold of 3 and discarding the first 30 frames of each time-stack. Following visual inspection, masks were interactively determined in order to limit analyses to membrane areas of neighboring cells. The programs detect the position of the blinking dye molecules, use a 2D Gaussian to calculate their position, and compile a matrix containing the signal amplitude, the x- and y- coordinates, and the corresponding errors. Based on this matrix, relative pairwise distance distribution histograms (0–200 nm) for Ripley´s structuring analysis [28], signal counts, and pointillistic images of the CLDN5 and ZO-1 stained junction areas between two endothelial cells were created.

### 4.8. Statistical Analysis

Experiments were performed at least 4 times in at least 3 replicates. Data are presented as mean ± standard deviation (SD) after being checked for normal distribution (Shapiro–Wilk test and graphically). For image analysis, at least 10 random areas per condition were analyzed (final *n* = 40). Histograms were created from 20 different spots per treatment condition. A two-sided student´s t-test was used for statistics, *p* < 0.05 was considered significant. 

## 5. Conclusions

In conclusion, combining different methods, like the Transwell monolayer TER monitoring and solute transport studies, with digital, total cell layer immunocytochemistry, and single molecule localization microscopy provides a multi-level approach which eliminates the background noise of inter-experimental variation and overcomes the limitations of each method by the provision of respective complementary information. Our novel experimental workflow should allow for more in-depth analyses in complex processes such as paracellular solute transport across TJ and the structural and functional modulation by respective compounds. 

## Figures and Tables

**Table 1:** Immunofluorescence intensity quantification (in arbitrary units; A.U. grey scale image fluorescence signal normalized to analyzed area) performed on maximum z-projection after incubation with 24 mM AlaGln for 24 h. Antibodies with different characteristics were used to quantify ZO-1 and CLDN5 abundance either on the whole image or limited to junction area at the cell-cell contact and resulted in similar findings for ZO-1, but not for CLDN5.

Quantification of Immunofluorescence Intensity.					
		ZO-1 (Conjugated)		CLDN5 (Unconjugated)	
		monoclonal	monoclonal	monoclonal	polyclonal
		555 nm	647 nm	555 nm	647 nm
Whole	Medium	0.32 (±0.16)	0.25 (±0.15)	0.11 (±0.06)	0.6 (±0.3)
	24 mM AlaGln	0.6 (±0.3)	0.59 (±0.19)	0.65 (±0.29)	0.6 (±0.3)
	*p*-value	**0.0013**	**0.005**	**0.001**	0.85
Junction	Medium	19 (±12)	20 (±12)	12.7 (±7.5)	30 (±19)
	24 mM AlaGln	35 (±20)	25 (±11)	29.5 (±13.6)	26 (±14)
	*p*-value	**0.048**	0.095	**<0.0001**	0.67

## Data Availability

Data is contained within the article and Supplementary Material.

## Supplementary Materials

The following are available online at https://www.mdpi.com/article/10.3390/ijms22158178/s1.
