# Supplementary material for: An Experimental Workflow for Studying Barrier Integrity, Permeability, and Tight Junction Composition and Localization in a Single Endothelial Cell Monolayer: Proof of Concept
Source: Int J Mol Sci. 2021 Jul 30;22(15):8178. doi: 10.3390/ijms22158178 (PMC8347178; doi:10.3390/ijms22158178)

Supplementary Materials: The following are available online at www.mdpi.

Figure S1: Cluster analysis of the co-localized ZO-1 and CLDN5 molecules. Ripley´s pairwise distance frequency histogram for 400 nm distance showing organized, non-random distances between ZO-1 – CLDN5 complexes. The frequencies are comparable for both conditions.

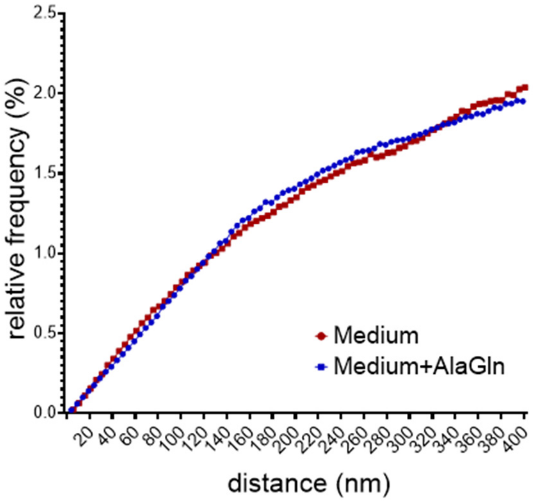

Supplement: Supplementary file 1 [file ijms-22-08178-s001.zip › ijms-1293763-supplementary.pdf]
